# Enhancing Medical Resident Confidence in Managing Anxiety and Depression on the Medical Floor Through Evidence-Based Guidelines

**DOI:** 10.1192/j.eurpsy.2025.2377

**Published:** 2025-08-26

**Authors:** J. Li, M. Khan, H. Raai

**Affiliations:** 1 Department of Psychiatry and Behavioral Health, SBH Health System, Bronx, United States

## Abstract

**Introduction:**

Medical and psychiatric conditions often coexist, with complex interrelationships that affect patient outcomes. Medical conditions can induce mental illness through psychological or physiological effects, while mental illness may hinder engagement in medical care due to barriers such as poor motivation and functional impairment. Anxiety and depression are among the most common psychiatric conditions encountered on medical floors, and improved management of these conditions can enhance patient outcomes and increase post-discharge follow-up.

**Objectives:**

The objective of this project was to improve the confidence of medical professionals in treating anxiety and depression on the medical floor by establishing an evidence-based approach for identifying and managing these conditions. The guidelines developed serve as a protocol for rapid, safe interventions, improving outcomes and reducing inefficient healthcare utilization.

**Methods:**

A survey was conducted among 18 medical residents to assess their confidence in evaluating and managing anxiety and depression, and determining when a psychiatric consult is necessary. After completing the initial survey, residents were provided with a stepwise guideline for managing anxiety and depression on the medical floor. A post-guideline survey was then conducted to assess changes in their confidence levels and understanding.

**Results:**

Pre-intervention survey results showed 85.7% of residents were comfortable identifying anxiety, 64.3% with identifying major depressive disorder (MDD), and 71.4% with differentiating MDD from bipolar depression. However, only 64.3% felt comfortable treating anxiety, and 50% initiating treatment for MDD. Post-guideline survey results showed improvements in several areas, including 90% confidence in identifying anxiety and 70.2% in diagnosing MDD. Notably, all residents felt comfortable continuing treatment for depression, and knowledge of medication side effects increased to 100%.

**Conclusions:**

The results suggest that medical residents on the floor showed improved confidence in evaluating and managing anxiety and depression after receiving the guideline. There was increased comfort with knowledge of medications and their side effects, although some discomfort remained in initiating treatment and titrating medications. Implementing universal screening protocols and further educational interventions, such as didactic sessions on specific medications and their side effects, can help address these gaps and enhance psychiatric care on the medical floor.

**Disclosure of Interest:**

None Declared

